# Information processing style and institutional trust as factors of COVID vaccine hesitancy

**DOI:** 10.1038/s41598-024-60788-y

**Published:** 2024-05-06

**Authors:** Wanchen Zhao, Catherine Maya Russell, Anastasia Jankovsky, Tyrone D. Cannon, Christopher Pittenger, Helen Pushkarskaya

**Affiliations:** 1 Department of Psychology, Yale University, 100 College St, New Haven, CT 06510 USA; 2 Department of Psychiatry, Yale School of Medicine, 34 Park Street, 3rd Floor, New Haven, CT 06519 USA; 3 Wu Tsai Institute, Yale University, New Haven, CT USA; 4 Yale Child Study Center, Yale School of Medicine, New Haven, CT USA; 5 Yale Center for Brain and Mind Health, Yale School of Medicine, New Haven, CT USA

**Keywords:** Psychology, Public health

## Abstract

This study investigates the factors contributing to COVID vaccine hesitancy. Vaccine hesitancy has commonly been attributed to susceptibility to misinformation and linked to particular socio-demographic factors and personality traits. We present a new perspective, emphasizing the interplay between individual cognitive styles and perceptions of public health institutions. In January 2020, before the COVID-19 pandemic, 318 participants underwent a comprehensive assessment, including self-report measures of personality and clinical characteristics, as well as a behavioral task that assessed information processing styles. During 2021, attitudes towards vaccines, scientists, and the CDC were measured at three time points (February–October). Panel data analysis and structural equation modeling revealed nuanced relationships between these measures and information processing styles over time. Trust in public health institutions, authoritarian submission, and lower information processing capabilities together contribute to vaccine acceptance. Information processing capacities influenced vaccination decisions independently from the trust level, but their impact was partially mediated by authoritarian tendencies. These findings underscore the multifactorial nature of vaccine hesitancy, which emerges as a product of interactions between individual cognitive styles and perceptions of public health institutions. This novel perspective provides valuable insights into the underlying mechanisms that drive this complex phenomenon.

## Introduction

Vaccination has played a crucial role in combating the COVID-19 pandemic and saving lives, estimated to have prevented 14.4 million deaths globally^1^. However, the decision to get vaccinated wasn't straightforward for many^2,3^. To understand them, consider a scenario: You're on a family road trip when a speeding car crashes into your SUV, leading to an Emergency Room visit. Faced with consent forms for a novel medical procedure you don’t fully understand, do you fully trust the ER doctors’ recommendations? Or try to contact your family doctor and franticly Google the meaning of the unfamiliar medical terms? This scenario mirrors the decisions many confronted during the pandemic peak, with normal lives interrupted^4^ and vaccines endorsed as the ultimate solution^5^. How would your attitudes toward the treatment change if you could delay it, join a support group, or seek second opinions? This extended scenario resembles how attitudes toward vaccination evolved, from initial availability to later, when vaccines were readily accessible and often mandated, and many vaccinated people shared their experiences.

Vaccine hesitancy, defined as the delay in acceptance or the refusal to get vaccinated, presented a unique challenge to the global recovery^6,7^, hampering the formation of herd immunity^8^. Prior research has linked vaccine hesitancy to the reduced perceived risk of COVID-19, the low perception of the vaccine safety, lower trust in public health institutions, right-wing politics, and tendencies toward conspiratorial beliefs^9–15^. Education, income, and population density have been linked to vaccine acceptance, while being Black, middle aged, and identified as women, the belief that truth is political, and susceptibility to online misinformation associated with vaccine refusal^11,12,16–22^. Elevated levels of paranoia triggered doubts about vaccine safety^23^, while anxiety about infection often outweighed concerns about side effects^24^. Some personality tendencies were linked to vaccination decisions. Elevated psychopathy, narcissism, and egoism increased vaccine refusal^16–20^. Different aspects of authoritarian tendencies, such as authoritarian aggression and obedience (authoritarian submission)^25–31^, impacted vaccination decisions differently. Obedience helps groups achieve goals during crises (e.g., form herd immunity during the pandemic)^32^, while authoritarian aggression erodes social trust^33^, including trust in public health institutions^34^.

Previous work has generally assumed that individuals kept autonomy over their vaccination decisions and examined how personal preferences are influenced by individual characteristics or circumstances. An alternative framework, drawn from democratic theory and empirical political science^35–37^, suggests that individuals in many social scenarios have conflicting preferences regarding retaining autonomy versus delegating decision to institutional authority or independent experts^38–41^. Despite the effort and depletion involved in the decision process, people often prefer to retain responsibility for choosing, even when they would benefit from others’ choice on their behalf^42–44^. However, there is also a well-documented reluctance among individuals to make irreversible choices^45–47^. Instead of maintaining autonomy over choosing between available options, individuals may prefer to delegate informational, moral, and other burdens to those they perceive as experts or authority figures^48^.

The preference for autonomy versus delegation reflects the interaction between an individual’s characteristics and the decision context. Delegation is more likely to occur when informational burden and potential losses are higher^45,48^. Men are more likely to retain decision autonomy than women do^49^, while individuals with higher authoritarian submission are more likely to demonstrate unquestioning obedience, relinquishing their decision to institutional authority^50,51^. As another crucial factor in delegation decisions^52–54^, trust increases the likelihood of transferring decision^55^. This effect is more pronounced with increasing technological sophistication, as trust helps offset the rising cost of decision-making^53^.

However, the key reason for delegating is to involve individuals with decision-relevant knowledge^56–58^ and to reduce the cost and effort required to obtain such expertise^59,60^. Complex decisions generate information processing needs that, at times, may surpass an individual's cognitive capacity, leading to information overload, which can result in lower decision quality and choice avoidance^58,60–65^. Choice avoidance may take the form of delegating decision^66^. Thus, information processing capacities impact the tendency to delegate. It remains unclear whether they also impact individual tendencies to trust those in authority. Unlike other forms of choice avoidance (delaying the choice, making small reversible steps, or choosing randomly), delegation allows transferring responsibility for choice outcome to another party. Thus, people are more likely to delegate to those with the status to assume responsibility, especially in situations in which this accountability manifests in a more careful decision^45,49^.

**Figure 1:**
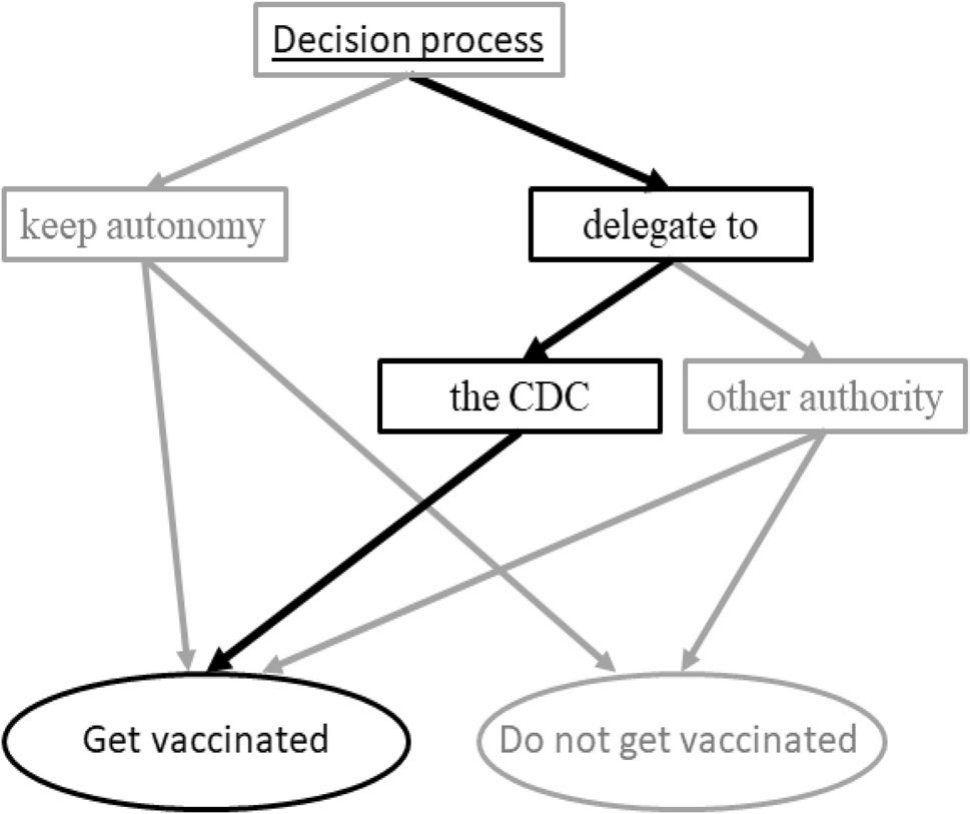
The vaccination decision framework that incorporates an option of delegating the decision rights.

The decision on COVID-19 vaccination required balancing the risks of illness and vaccine side effects, along with the potential of spreading infection, amidst changing and conflicting information. Consequently, many opted to defer this decision to an expert authority. Institutional authority on vaccination decision during the COVID-19 pandemic in the U.S. was assigned to the Centers for Disease Control and Prevention (CDC), who had responsibility to share best available health information and recommendations with clinicians and the general population^67–70^. The public evaluated this information and made a choice; one option was to simply follow the CDC recommendations. The global scientific community took actions to reassure general public on the robustness of the scientific review and approval of COVID-19 vaccines, supporting authority of the CDC^71,72^. Many others claimed to have an expertise in COVID-19 vaccines, although, not necessarily offering to take responsibility for the choice outcome. A broad range of opinions was promoted, both supporting and challenging the CDC authority^73,74^. Thus, we suggest that the COVID-19 vaccination decision could be modeled as a choice between (1) retaining autonomy over the decision and assessing the available information to determine if vaccination is the best choice for one's personal situation, (2) delegating the decision to CDC and adhering to its recommendation for vaccination, or (3) following recommendations of other authorities (Fig. 1).

In 2021, we surveyed 318 individuals from the general population during three phases of the vaccination program: (1) February 20th–March 10th, when the vaccine rollout had just begun; (2) April 20th–May 10th, following the first reports of allergic reactions to the vaccine; and (3) September 20th–October 20th, when vaccines were broadly available and often mandated. We aimed to examine the evolution of perceptions regarding vaccine safety and the decision to get vaccinated within the context of our decision model (Fig. 1). Note that our model does not explicitly address the difference between recommendations and mandates from the authorities. Thus, we anticipate that some of the predicted effects (detailed below) may differ in wave 3, when mandates were more common.

Directly assessing whether individuals kept autonomy or delegated their decision, and if so, whose advice they chose to follow, is not feasible using self-reports (e.g., due to reporting bias and the lack of insight). However, our model makes several predictions that can be tested with these data. Given the central role of the CDC in directing public health responses to health crises, we primarily focused on factors that may influence the likelihood of delegating decision to the CDC. The model predicts that higher tendency to trust the CDC and the scientific community, and higher authoritarian submission would positively associate with the likelihood to follow the CDC’s recommendations to get vaccinated. Trust in the CDC and science can be assessed by asking participants directly. Authoritarian value orientation is a multidimensional concept that can be assessed using the F-scale^75^. Prior research used several versions of the F-scale^25–31^. When authoritarian tendencies were not a focus of the investigation but were used as covariates (as we aimed here), some studies used a subset of items from the F-scale to reduce the burden on study participants^33,76–78^. We employ the same approach (details and limitations are discussed in Methods).

The model predicts that lower information processing capabilities will be associated with a tendency to delegate decision, reflecting choice avoidance. Testing this prediction is the key focus of this study. We evaluated participants’ information processing styles using a behavioral task, the Perceptual and Value-based Decision Making (PVDM^79^). Because some information processing may occur outside of awareness^80^, computational modeling of the data from behavioral tasks, such as PVDM, can better quantify variations in information processing than self-reports^81–83^ or descriptive measures of behavior^84^. The Drift–Diffusion Model of choice (DDM)^85,86^ is one robust framework for such modeling. According to DDM, a choice is made only when the evidence favoring one option crosses a critical threshold. Higher decision thresholds lead to more accurate, cautious, and slower decisions, indicating a need for more supporting evidence. Another DDM parameter, the drift rate, reflects the effectiveness of evidence accumulation. It positively correlates with cognitive ability^87^ and negatively with doubt and indecisiveness^88^. The drift rate tends to decrease during more difficult decisions^85^, which illustrates that, in contrast to intelligence, it is not just a trait-level characteristic but is influenced by decision context.

The decision threshold and the drift rate characterize individual information processing style. They generally only provide information on *how* a decision is formed and not on *what* decision is made. However, our model allows some predictions. For instance, higher decision thresholds may be linked to a tendency to delay the vaccination decision until more information is available. Low drift rates reflect reduced information processing capacity, which we predicted to positively associate with delegating the decision to authority.

Previous studies have shown that heightened levels of anxiety and paranoia can influence perceptions of vaccine safety and decisions regarding vaccination^23,24^. These observed effects may stem from impaired information processing in these conditions^89,90^. The drift rate is reduced in paranoia and anxiety, which may contribute to impairment in such processes^91,92^. However, other factors, such as altered belief updating and negativity bias, also play significant role^15,93,94^. Thus, the impact of reduced drift rates on the tendency to delegate decisions to the CDC may diverge in individuals with elevated levels of paranoia and anxiety.

The PVDM task characterizes individual information processing style in four decision contexts: during perceptual decisions and value-based judgments^79^, both during a neutral condition and following random negative feedback (to induce negative affect^95–97^). One of the four experimental conditions, value-based decision making following random negative feedback, probes how individuals form value-based subjective judgements when influenced by negative emotions. Negative emotions and stress can significantly alter decision making processes ^98,99^. Given the increase in negative affect in general public during COVID-19 pandemic^100,101^, we anticipated that information processing style during this condition is predictive of how the participants processed information about COVID-19 vaccine at the peak of the pandemic.

Our model suggests that individuals could choose to follow advice from other individuals and organizations, which might support or oppose the CDC guidance. While trust in CDC and science predict individual tendencies to delegate decision to the CDC, tendency for obedience and lower information processing capacities predict the tendency to relinquish the decision to authority more generally, including to those supporting and opposing the CDC recommendations. This may manifest in elevated noise in how these tendencies are associated with the likelihood of getting vaccinated, reducing sensitivity of our approach.

We tested our predictions in two steps. First, we employed panel data analyses to examine longitudinal influences on perceptions of vaccines’ safety and vaccination decision. Second, we employed structural equation modeling to test whether the hypothesized effects of information processing styles on vaccination decisions are independent of or mediated by confidence in the CDC and the scientific community. For this second test, we used data collected between September 20th and October 20th, when access to the vaccine was no longer limiting. We discuss policy implications of our results in the context of promoting positive interactions between individuals and public health institutions.

## Results

*Descriptive statistics* Participant characteristics are reported in Table 1. Bivariate relations among all variables and longitudinal changes in variables assessed at multiple phases of the pandemic are reported in Supplementary Information (SI1).

**Table 1:** Participant characteristics.

	Mean (SD) or N (%)	Median or Percentage in the U.S. (Census 2019)
Control variables		
Gender (1 = “male participant (M)”/ 0 = “female participant (F)”)	156 (49.06)/165 (50.94)	49.5% (M)/50.5% (F)
Race (1 = “African American”/0 = “non-African American”)	26 (8.18)/292 (91.82)	12.4% African American
Ethnicity (1 = “Hispanic”/0 = “non-Hispanic”)	21 (6.60)/296 (93.8)	18.7% Hispanic
Age (years)	38.83 (11.95)	38.8
Education level		
0 = “No college degree”	104 (32.7)	54.4%
1 = “College”	132 (41.5)	21.2%
2 = “Higher than college”	82 (25.8)	13.8%
Income		
1 = “5000–14,999”	26 (8.2)	9.3%
2 = “15,000–19,999”	30 (9.4)	6.3%
3 = “20,000–24,999”	15 (4.7)	6.5%
4 = “25,000–29,999”	17 (5.4)	6.0%
5 = “30,000–39,999”	20 (6.3)	11.2%
6 = “40,000–49,999”	40 (12.6)	9.5%
7 = “50,000–59,999”	34 (11.0)	8.2%
8 = “60,000–69,999”	29 (9.1)	7.1%
9 = “70,000–79,999”	29 (9.1)	5.7%
10 = “80,000–89,999”	23 (7.2)	2.5%
11 = “90,000–99,999”	12 (3.8)	3.6%
12 = “100,000–149,999”	8 (2.5)	9.9%
13 = “150,000–199,999”	19 (6.0)	3.2%
14 = “200,000 < ”	7 (2.2)	1.5%
State population density (people per square mile) in 2021		
Less than 50	30 (9.4)	Less than 100–19%
50–99	28 (8.8)	
100–149	30 (9.4)	More than 100–81%
150–199	29 (9.1)	
200–249	22 (6.9)	
250 ≤	178 (56.0)	
State legislature in 2021		
1 = “predominantly Democratic”	159 (50.0)	48.0%
−1 = “predominantly Republican”	110 (34.6)	29.9%
0 = “equally Split”	48 (15.1)	15.1%
Unemployed because of COVID (1 = “Yes”/0 = “No”)	5 (0.02)/275 (86.79)	
COVID in family (1 = “Yes”/0 = “No”)	46 (14.47)/234 (73.58)	
Variables influencing tendency to delegate the decision		
F-score	17.45 (7.02)	
Trust in CDC	3.87 (1.36)	
Trust in social media	1.89 (0.84)	
Belief in science	3.75 (1.03)	
Perceived knowledge of COVID	1.03 (0.80)	
External locus of control	3.30 (1.24)	
Internal locus of control	4.73 (0.85)	
Variables influencing tendency to avoid choice (information processing capacity)		
Behavior-based measures during value-based choices following random negative feedback		
Decision boundary	2.03 (0.43)	
Drift rate	1.23 (0.30)	
Non-decision time	0.62 (0.16)	
Clinical measures (online only subsample)		
BSI—Elevated level of anxiety (1 = “Yes”/0 = “No”) BSI—Elevated level of paranoia (1 = “Yes”/0 = “No”)	41 (15.65)/218 (83.20) 61 (23.28)/198 (75.57)	

Descriptive statistics of study sample, with comparison between demographic information and national median.

*Panel data analyses* Not surprisingly, perceptions of vaccine safety were higher, across waves, among individuals who received one or more COVID vaccinations (Cohen’s d were 0.61, 0.92, 1.30 for waves 1, 2, and 3, respectively; *ps* < 0.001). To avoid multicollinearity, we examined longitudinal determinants of perceptions of vaccine safety and the choice to get vaccinated separately (Figure SI2). Model fit indices are provided in Table S2-1 for models with vaccination status as the dependent variable and in Table S2-2 for models with perceived safety as the dependent variable. Because the decision to vaccinate is irreversible, we included the lagged vaccination status as a predictor (see Methods). This allowed us to examine the effects of the remaining variables on the decision to get vaccinated at each time point, independent of prior vaccination status.

Table 2 illustrates that we replicated many previous results (*base models*). Later stage of the pandemic (September–October 2021), older age, being in low or in high income classes (U-shaped relations with income), state population density, and unemployment because of COVID predicted higher likelihood of getting vaccinated. Higher likelihood of deciding to get vaccinated during later stages of the pandemic may reflect both higher acceptance of the vaccines and impacts of vaccine mandates. Perception of vaccine safety was lower during early stages of the vaccination program (February–March 2021) and in individuals with an authoritarian value orientation. Education level, belief in science competence, and trust in the CDC were positively associated with both perception of vaccine safety and decision to get vaccinated. Black participants viewed the vaccine as less safe (*β* = − 0.41 *p* = 0.07) and were less likely to get vaccinated (*OR* 0.35, *p* = 0.045).

**Table 2:** Panel linear model statistic for full sample.

	DV = Vaccinated				DV = Perceived safety			
	Base	Model 1	Model 2	Model 2 (short)	Base	Model 1	Model 2	Model 2 (short)
Constant	− 5.00**** **(1.23)**	− 3.81*** **(1.30)**	− 2.47* (1.47)	− 2.67** **(1.19)**	3.02**** **(0.67)**	3.78**** **(0.82)**	4.29**** **(0.94)**	4.54**** **(0.66)**
Lagged vaccination	4.80**** **(1.09)**	4.81**** **(1.09)**	5.26**** **(1.31)**	5.08**** **(1.21)**				
Wave 1	− 1.98**** **(0.48)**	− 2.01**** **(0.48)**	− 3.29* (1.72)	− 2.18* (1.30)	− 2.70*** **(1.06)**	− 2.62*** **(1.04)**	− 3.85** **(1.98)**	− 4.52**** **(1.30)**
Wave 3	1.07** **(0.51)**	1.07** **(0.51)**	− 2.22* (1.21)	− 0.57 (0.76)	− 0.89* (0.47)	− 0.87* (0.47)	− 1.92*** **(0.77)**	− 1.28*** **(0.52)**
Control variables								
Gender (Male participant)	− 0.23 (0.22)	− 0.18 (0.24)	0.02 (0.30)	0.10 (0.27)	0.17 (0.13)	0.17 (0.13)	0.16 (0.12)	0.17 (0.13)
Age ≤ 35	0.39 (0.25)	0.34 (0.26)	0.42 (0.29)	0.40 (0.27)	− 0.07 (0.14)	− 0.12 (0.14)	− 0.11 (0.13)	− 0.07 (0.13)
Age ≥ 60	1.86*** **(0.60)**	1.85*** **(0.60)**	1.82*** **(0.67)**	1.79*** **(0.61)**	− 0.06 (0.28)	− 0.03 (0.28)	− 0.01 (0.27)	− 0.06 (0.27)
Race (Black)	− 0.56 (0.36)	− 0.60* *(0.38)*	− 1.05** **(0.52)**	− 0.61^a^ (0.38)	− 0.39* (0.23)	− 0.42* (0.23)	− 0.41* (0.22)	− 0.40* (0.23)
Ethnicity (Hispanic)	0.59 (0.44)	0.52 (0.45)	0.22 (0.49)		0.06 (0.26)	0.00 (0.26)	0.05 (0.25)	
Education	0.43** **(0.18)**	0.44*** **(0.18)**	0.48** **(0.20)**	0.46*** **(0.18)**	0.22** **(0.09)**	0.22*** **(0.09)**	− 0.36 (0.42)	0.22*** **(0.09)**
Income	− 0.21* (0.11)	− 0.21* (0.11)	− 0.45** **(0.20)**	− 0.22* (0.12)	− 0.02 (0.06)	− 0.03 (0.06)	− 0.04 (0.06)	− 0.03 (0.06)
Income^2^	0.02** **(0.01)**	0.02** **(0.01)**	0.04** **(0.01)**	0.02** **(0.01)**	0.00 (0.00)	0.00 (0.00)	0.00 (0.00)	0.00 (0.00)
COVID in family	0.00 (0.25)	− 0.02 (0.25)	− 0.03 (0.27)		0.03 (0.11)	0.03 (0.12)	0.13 (0.15)	
Unemployed because of COVID	1.13** **(0.57)**	1.12** **(0.57)**	1.17** **(0.60)**		0.07 (0.31)	0.05 (0.31)	0.00 (0.31)	
State population density	0.11* (0.07)	0.11 (0.07)	0.15** **(0.08)**	0.18*** **(0.07)**	0.01 (0.04)	0.01 (0.04)	0.01 (0.04)	0.00 (0.03)
State politics	0.15 (0.15)	0.17 (0.15)	0.27 (0.17)		− 0.06 (0.08)	− 0.06 (− 0.08)	− 0.06 (0.08)	
Variables influencing tendency to delegate the decision								
Knowledge about COVID	0.23* (0.13)	0.23* (0.13)	0.20 (0.14)		0.09 (0.06)	0.09 (0.06)	0.09 (0.07)	
Science knows COVID	0.34*** **(0.13)**	0.35*** **(0.13)**	0.40*** **(0.15)**	0.40*** **(0.16)**	0.34**** **(0.05)**	0.34**** **(0.05)**	0.30**** **(0.06)**	0.28**** **(0.06)**
Trust in CDC	0.59**** **(0.14)**	0.57**** **(0.14)**	0.38*** **(0.15)**	0.39*** **(0.15)**	0.54**** **(0.05)**	0.54**** **(0.05)**	0.50**** **(0.07)**	0.48**** **(0.06)**
Trust in social media	0.19 (0.14)	0.17 (0.14)	0.19 (0.16)		0.06 (0.08)	0.04 (0.08)	0.03 (0.08)	
F-scale	0.00 (0.02)	0.00 (0.02)	− 0.03 (0.02)	− 0.03 (0.02)	− 0.06**** **(0.01)**	− 0.06**** **(0.01)**	− 0.06**** **(0.01)**	− 0.06**** **(0.01)**
Internal locus of control	− 0.05 (0.12)	− 0.06 (0.13)	− 0.12 (0.17)		0.06 (0.08)	0.06 (0.08)	0.10 (0.09)	
External locus of control	0.03 (0.09)	0.01 (0.09)	0.09 (0.12)		0.01 (0.06)	0.00 (0.06)	0.00 (0.05)	
Variables influencing tendency to avoid choice (information processing capacity)								
Behavior-based measures of information processing								
Decision boundary		− 0.14 (0.25)	− 0.14 (0.26)			− 0.02 (0.15)	− 0.04 (0.15)	
Drift rate		− 0.62* (0.37)	− 0.69* (0.40)	− 0.76** **(0.36)**		− 0.23 (0.23)	− 0.24 (0.22)	
Non-decision time		0.26 (0.68)	0.42 (0.74)			− 0.47 (0.43)	− 0.41 (0.42)	
Interactions by waves								
Gender x wave 1			− 0.73 (1.24)	− 0.82* (0.44)				0.12 (0.28)
Black x wave 1			1.24 (0.84)					
Income x wave 1			0.31 (0.24)					
Income^2^ x wave 1			− 0.02 (0.02)					
F-scale x wave 1			0.06 (0.04)	0.06** **(0.03)**				0.02 (0.02)
LOC-internal x wave 1			0.20 (0.27)					
LOC-external x wave 1			− 0.32 (0.21)					
Hispanic x wave 3			1.84 (1.14)					
Income x wave 3			0.50 (0.33)					
Income^2^ x wave 3			− 0.03 (0.03)					
Education x wave 1							1.69 (1.31)	
COVID in family x wave 1							− 0.52 (0.47)	
Science knows COVID x wave 1			0.47* (0.24)	− 0.16 (0.24)			0.34* (0.18)	0.39** **(0.16)**
Science knows COVID x wave 3			0.47* (0.24)	0.43** **(0.22)**			0.11 (0.07)	0.10 (0.06)
Rho	1.38E−16 (5.82E + 07)	1.62E−07 (8.65E + 06)	1.38E−16 (1.62 + 07)	3.05E−16 (7.73E + 06)				

Predictors for vaccination status and perceived vaccine safety. Statistics for DV = vaccination were derived from logistic panel regression with random effects; D = safety from panel linear regression with random effects. Results were averaged from 5 imputations. ^a^—*p* = 0.11, *—*p* < 0.1, **—*p* < 0.05, ***—*p* < 0.01, ****—*p* < 0.001.

Significance levels with *p* < 0.11 are in [bold].

Our key novel result (*models 1–2*) is that drift rate (i.e., effectiveness of information processing) during value-based judgements following random negative feedback was associated with lower likelihood of getting vaccinated (*β* = -0.76 *p* = 0.03). This supports the prediction that individuals with reduced information processing capabilities would delegate decision to the CDC, reflecting choice avoidance. The DDM parameters from the remaining PVDM conditions (perceptual choices and value-based choices without the feedback) did not significantly relate to perceptions of vaccine safety or the decision to get vaccinated (*ps* > 0.18), confirming that information processing styles are context dependent.

Further analysis (*model 2*) revealed that the impact of some variables varied across stages of the vaccination program. Belief in science competence impacted perception of vaccine safety more strongly during earlier stages (February–March 2021) and vaccination actions during later stages of the vaccination program (September–October 2021). This may reflect stronger vaccine hesitancy among committed “science deniers.” Contrary to prior studies but consistent with predictions of our model, male participants were somewhat less likely to get vaccinated than female participants during earlier stages of the vaccination program (*OR* 0.44, *p* = 0.062), consistent with a reduced tendency to delegate decision in men. Consistent with prior studies, an authoritarian value orientation was negatively associated with perceptions of vaccine safety; however, consistent with the predictions of our model, it was positively associated with the likelihood of getting vaccinated early in the vaccination program, perhaps reflecting a tendency to relinquish decision to an institutional authority.

**Figure 2:**
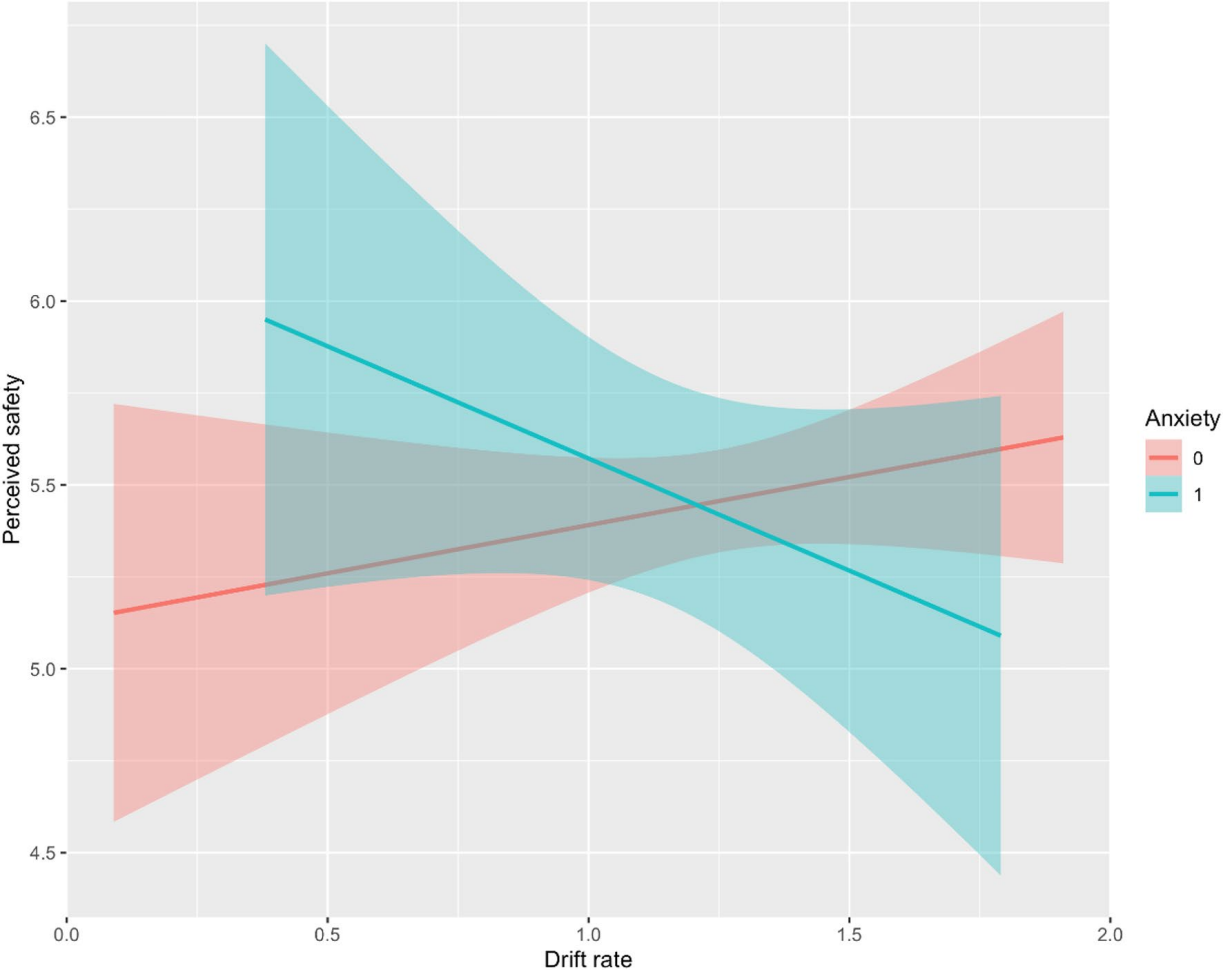
Clinical level of anxiety by drift rate interaction. Anxiety by drift rate (on value-based decision-making following emotional triggers) interaction. The perceived vaccine safety of participants with elevated anxiety were negatively affected by drift rate—higher perceived safety with low drift rate, but with higher drift rate they exhibit similar perceived safety as low-anxiety participants.

Next, we examined the impact of elevated anxiety and paranoia, which may impact information processing style. This was done using subsample of participants who completed the Brief Symptom Inventory (N = 262). We replicated most relations seen in the full sample (see Table 3) and, additionally, replicated the finding that individuals with elevated paranoia perceived vaccination as less safe. A novel result was that, in individuals with elevated anxiety, lower drift rates were associated with higher perceived safety of the vaccine, while in all other individuals this relationship remained non-significant (see Fig. 2).

**Table 3:** Panel linear model statistic for online sub-sample.

	DV = Vaccinated			DV = Perceived safety		
	Model 2, short	Model 3	Model 4	Model 2, short	Model 3	Model 4
Constant	− 2.89** **(1.48)**	− 3.17** **(1.53)**	− 3.03** **(1.55)**	4.66**** **(0.75)**	3.51**** **(0.76)**	3.55**** **(0.71)**
Lagged vaccinatio n	5.17**** **(1.26)**	5.16**** **(1.26)**	5.10**** **(1.25)**			
Wave 1	− 1.64 (1.67)	− 1.56 (1.68)	− 1.52 (1.73)	− 4.37**** **(1.43)**	− 4.31**** **(1.39)**	− 4.25**** **(1.30)**
Wave 3	− 1.24 (0.97)	− 1.22 (0.98)	− 1.15 (1.00)	− 1.45*** **(0.85)**	− 1.29** **(0.54)**	− 1.23** **(0.51)**
Control variables						
Gender (Male participant)	0.09 (0.30)	0.18 (0.30)	0.15 (0.31)	0.15 (0.15)	0.11 (0.13)	0.07 (0.13)
Age ≤ 35	0.30 (0.30)	0.49 (0.31)	0.51 (0.32)	− 0.04 (0.15)	0.02 (0.13)	0.03 (0.13)
Age ≥ 60	1.71** **(0.74)**	1.76** **(0.75)**	1.67** **(0.76)**	− 0.13 (0.31)	− 0.04 (0.27)	− 0.13 (0.28)
Race (Black)	− 0.44 (0.44)	− 0.42 (0.45)	− 0.43 (0.56)	− 0.18 (0.26)	− 0.24 (0.22)	− 0.23 (0.23)
Education	0.58*** **(0.22)**	0.61*** **(0.22)**	0.62*** **(0.22)**	0.24** **(0.10)**	0.21** **(0.09)**	0.23*** **(0.09)**
Income	− 0.21 (0.15)	− 0.26 (0.16)	− 0.25 (0.16)	− 0.07 (0.07)	− 0.07 (0.06)	− 0.08 (0.06)
Income^2^	0.02 (0.01)	0.02* (0.01)	0.02* (0.01)	0.00 (0.00)	0.00 (0.00)	0.01 (0.00)
State population density	0.14* (0.07)	0.16** **(0.08)**	0.16** **(0.08)**	0.01 (0.04)	0.01 (0.03)	0.01 (0.03)
Variables influencing tendency to delegate the decision						
Science knows COVID vaccine	0.54*** **(0.21)**	0.53*** **(0.22)**	0.54*** **(0.21)**	0.25**** **(0.06)**	0.31**** **(0.06)**	0.26**** **(0.06)**
Trust in CDC	0.35** **(0.16)**	0.36** **(0.16)**	0.37** **(0.17)**	0.44**** **(0.07)**	0.40**** **(0.07)**	0.40**** **(0.06)**
F-scale	− 0.03 (0.02)	− 0.03 (0.03)	− 0.03 (0.02)	− 0.06**** **(0.01)**	− 0.05**** **(0.01)**	− 0.06**** **(0.01)**
Variables influencing tendency to avoid choice (information processing capacity)						
Behavior-based measures of information processing						
VLF (drift rate)	− 0.91** **(0.46)**	− 0.77 (0.48)	− 0.93 (0.57)	− 0.81 (0.24)	− 0.17 (0.21)	− 0.02 (0.25)
Clinical measures that may impact information processing						
Clinical level of anxiety		0.26 (0.39)	1.15 (1.76)		0.28 (0.18)	1.78*** **(0.72)**
Clinical level of paranoia		− 0.23 (0.33)	− 1.44 (1.61)		− 0.34** **(0.16)**	− 0.81 (0.64)
Interactions by waves						
Gender x wave 1	− 0.76 (0.50)	− 0.83 (0.51)	− 0.82 (0.51)	0.18 (0.30)	0.34 (0.37)	0.31 (0.29)
Science knows x wave 1	− 0.26 (0.32)	− 0.27 (0.33)	− 0.28 (0.33)	0.40** **(0.17)**	0.38** **(0.19)**	0.38*** **(0.16)**
Science knows x wave 3	0.64** **(0.29)**	0.66** **(0.30)**	0.65** **(0.30)**	0.14** **(0.07)**	0.13 (0.08)	0.12* (0.06)
F-scale x wave 1	0.06 (0.04)	0.06 (0.04)	0.05 (0.04)	0.00 (0.02)	0.01 (0.03)	0.01 (0.02)
Clinical measures by drift rate						
Anxiety x VLF			− 0.72 (1.34)			− 1.26** **(0.59)**
Paranoia x VLF			1.01 (1.26)			0.37 (0.51)

Predictors of vaccination status and perceived vaccine safety with online subsample, which measures anxiety and paranoia. ^a^—*p* = 0.11, *—*p* < 0.1, **—*p* < 0.05, ***—*p* < 0.01, ****—*p* < 0.001.

Significance levels with *p* < 0.11 are in [bold].

**Figure 3:**
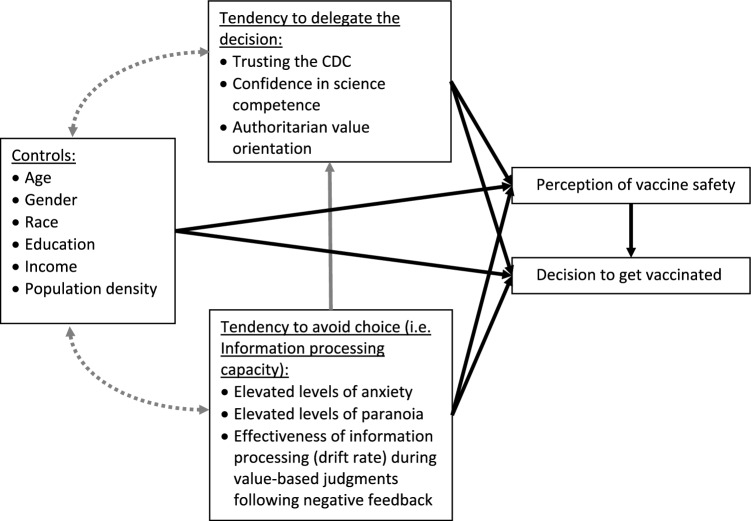
Conceptual model of direct and indirect effects tested during SEM analysis. *Note:* Dotted lines—covariances, are reported in Supplementary Materials. Solid lines—direct effects; effects depicted by black solid lines are reported in Table 4, effects depicted by the gray solid line are reported in Table 5.

*Structural equation modeling* (SEM) allows including both perception of vaccine safety and the vaccination decision in the same model; it handles the multicollinearity concerns by separating direct effects of predictors of the vaccination decision from the indirect effects of these predictors mediated through perception of vaccine safety. We examined the impact of three groups of factors: (1) factors that associate with trust toward public health institutions during the pandemic, (2) factors that influence information processing style, and (3) sociodemographic factors as control covariates (see Fig. 3). This analysis also allowed testing whether information processing style influenced vaccination decisions independently from or mediated by attitudes toward public health institutions. Significant direct and indirect relations, as well as model fit indices, are reported in Tables 4 and 5. The strongest predictor of the likelihood of getting vaccinated was perception of vaccine safety. Education level and belief in science competence affected vaccination choices only indirectly through positive association with perception of vaccine safety. However, younger age (≤ 35 years) and older age (≥ 60 years) were directly associated with higher likelihood of getting vaccinated. Being Black had a negative effect on the likelihood of getting vaccinated. Trust in the CDC predicted higher likelihood of getting vaccinated both directly and indirectly through higher perceived vaccine safety. Elevated levels of paranoia were indirectly linked to lower perceived vaccine safety (*β*_*std*_ = − 0.09, *p* = 0.05) through reduced trust in the CDC (*β*_*std*_ = − 0.14, *p* = 0.03).

**Table 4:** Structural equation modeling: standardized effects on perception of vaccine safety and vaccination decision.

	DV = Perception of vaccine safety			DV = Vaccinate		
	Direct	Indirect	Total	Direct	Indirect	Total
Perception of vaccine safety	–	–	–	0.59**** **(0.08)**	–	0.59**** **(0.08)**
*Control variables*						
Gender (Male participant)	0.06 (0.04)	–	0.06 (0.04)	− 0.02 (0.04)	0.04 (0.03)	0.01 (0.05)
Age ≤ 35 years	− 0.03 (0.05)	–	− 0.03 (0.05)	0.11** **(0.05)**	− 0.02 (0.03)	0.10 (0.05)
Age ≥ 60 years	0.002 (0.04)	–	0.002 (0.04)	0.15**** **(0.04)**	0.001 (0.02)	0.15**** **(0.05)**
Black	− 0.02 (0.04)	–	− 0.02 (0.04)	− 0.05 (0.06)	− 0.01 (0.03)	− 0.06 (0.05)
Education level	0.10** **(0.05)**	–	0.10** **(0.05)**	0.07 (0.05)	0.06** **(0.03)**	0.13** **(0.06)**
Income level	− 0.25* **(0.14)**	–	− 0.25* **(0.14)**	− 0.03 (0.13)	− 0.15* **(0.09)**	− 0.18 (0.16)
Income level ^2^	**0.21**^**a**^ **(0.13)**	–	**0.21**^**a**^ **(0.13)**	0.12 (0.14)	**0.13**^**a**^ **(0.08)**	0.24* **(0.16)**
State population density	0.08* **(0.05)**	–	0.08* **(0.05)**	0.07* **(0.04)**	0.05* **(0.03)**	0.12** **(0.05)**
Variables influencing tendency to delegate the decision						
Perceived level of knowledge of science	0.24**** **(0.06)**	–	0.24**** **(0.06)**	− **0.01** **(0.06)**	0.14**** **(0.04)**	0.13** **(0.06)**
Trust the CDC	0.45**** **(0.07)**	–	0.45**** **(0.07)**	0.19*** **(0.07)**	0.27**** **(0.05)**	0.46**** **(0.07)**
F-scale (authoritarian orientation)	− 0.23**** **(0.05)**	–	− 0.23**** **(0.05)**	0.09* **(0.05)**	− 0.14**** **(0.03)**	− 0.04 (0.05)
Variables influencing tendency to avoid choice (information processing capacity)						
Clinical measures						
Clinical level of anxiety	0.02 (0.05)	0.04 (0.04)	0.06 (0.07)	− 0.01 (0.04)	0.05 (0.05)	0.04 (0.06)
Clinical level of paranoia	− 0.01 (0.06)	− 0.09** **(0.04)**	− 0.10 (0.07)	0.02 (0.05)	− 0.08* **(0.05)**	− 0.06 (0.07)
Behavior-based measures of information processing						
Drift rate, value-based choices following emotional triggers	− 0.02 (0.05)	− 0.005 (0.04)	− 0.02 (0.06)	− 0.08* **(0.05)**	− 0.04 (0.04)	− 0.12** **(0.06)**

Statistics from structural equation modeling. ^a^—*p* = 0.11, *—*p* < 0.1, **—*p* < 0.05, ***—*p* < 0.01, ****—*p* < 0.001; N = 272; Model fit: X^2^(1) = 0.32 *p* = 0.57 CMIN/DF = 0.32; Bootstrap K = 2000; RMSEA < 0.01 with PCLOSE = 0.68. Indirect effect of paranoia on perception of vaccine safety is through decreased trust in CDC message: Direct effect on trust in CDC β_std_ = − 0.14 S.E = 0.07 *p* = 0.05.

Significance levels with *p* < 0.11 are in [bold].

**Table 5:** Structural equation modeling: standardized direct effects of clinical and behavior-based measures on self-reports of subjective beliefs.

	DV = F-scale	DV = Level of knowledge of science	DV = Trust CDC
Drift rate, value-based choices following negative feedback	− 0.14** **(0.06)**	− 0.01 (0.06)	− 0.08 (0.06)
Clinical level of anxiety	− 0.02 (0.07)	− 0.02 (0.06)	0.09 (0.07)
Clinical level of paranoia	0.10 (0.06)	− 0.02 (0.06)	− 0.14** **(0.07)**

^a^—*p* = 0.11, *—*p* < 0.1, **—*p* < 0.05, ***—*p* < 0.01, ****—*p* < 0.001.

Significance levels with *p* < 0.11 are in [bold].

The direct impact of the drift rate from value-based choices following negative feedback on the likelihood of getting vaccinated was negative (*β*_*std*_ = − 0.08, *p* = 0.10), and the total effect was even more negative (*β*_*std*_ = − 0.12, *p* = 0.05). The additional indirect negative premium was most likely driven by the reduced drift rates in individuals with higher scores on F-scale (*β*_*std*_ = − 0.14, *p* = 0.019), who were more likely to comply with the CDC guidance.

Authoritarian value (F-score) had a negative direct effect (*β*_*std*_ = − 0.23, *p* < 0.001) on the perception of vaccine safety but a positive direct effect (*β*_*std*_ = 0.09, *p* = 0.052) on the likelihood of getting vaccinated. All 5 items from the F-scale had high loadings (≥ 0.615) on a single factor (principle axes factoring with Promax rotation with Kaiser normalization^102^), indicating that together they measure the same construct (SI3, Table S3-1); however, this construct is thought to be multidimensional^25–30^. To explore the contributions of different dimensions of authoritarian personality, we ran a second SEM with the F-score broken down into 5 individual items (Table S4); this revealed that different F-scale items related differently to vaccine perceptions and actions. Q2 (superstition) and Q5 (authoritarian aggression) negatively related to vaccination likelihood only through their direct negative effect on the perception of vaccine safety; this clarifies previously reported reduced perception of vaccine safety in these individuals. Q3 (authoritarian submission), on the other hand, directly and positively related to vaccination likelihood; this was predicted by our model. The negative relationship between the drift rates and the authoritarian tendencies was primarily driven by negative associations with authoritarian submission (probed by Q1 & Q3, see Table S3-2) and authoritarian aggression (probed by Q5). These patterns need to be replicated in studies that use the full F-scale.

## Discussion

Using longitudinal data, we examined determinants of perceptions of vaccine safety and the decision to get vaccinated across different stages of the COVID vaccination program (from February to October 2021). Guided by a framework, drawn from democratic theory and empirical political science^35–37^, we considered the vaccination decision in the context of conflicting preferences regarding retaining autonomy over individual choices and delegating decision to institutional authority^38–41^. The Public Health Service Act of 1944 (PHSA)^103^ authorizes the secretary of the Department of Health and Human Services to take regulatory action to prevent the introduction and spread of infectious diseases; this authority has subsequently been delegated to the CDC^104^. This delegation provides the CDC with both robust authority and responsibility to implement science-based public health measures during public health crises. Thus, we specifically focused on factors that may impact the tendency in general public to delegate decision to the CDC and follow their recommendation to get vaccinated. We find that factors that are predicted to associate with a tendency to delegate decision to authority were positively associated with the likelihood of getting vaccinated. These factors include trusting the CDC and scientific community, a tendency toward obedience, and lower information processing capacities in the high stress affective contexts—such as the pandemic.

Higher trust in the CDC and scientific community have been linked to likelihood to get vaccine by many previous studies. The link between reduced drift rates and vaccination decisions shown here is novel; it aligns with theoretical predictions that lower information processing capacities may lead to choice avoidance and manifest as delegating choices to authority.

We find that individuals with pre-pandemic elevated anxiety and less effective information processing tended to see the vaccine as safe, while those with anxiety and more effective information processing viewed the vaccine as less safe. This result may reflect a tendency among individuals with elevated anxiety and high information processing capacities to retain the decision autonomy and at the same time, due to negativity bias^94^, to be more sensitive to negative information about the vaccines. Elevated levels of paranoia did not modulate relations between drift rates and vaccination decisions. As in prior results^15,105,106^ it was associated with less positive perception of vaccine safety. This negative impact was fully mediated through reduced trust in the CDC. Paranoia has long been associated with difficulties developing trust that lead to pervasive interpersonal challenges^107^. Our results extend this dynamic to institutions: lack of trust in public institutions may generate additional social challenges for these individuals.

We find that superstition and authoritarian aggression were negatively related to self-reported perception of vaccine safety, while authoritarian submission may be linked to a tendency to relinquish the decision to an institutional authority. The drift rates were negatively associated with authoritarian tendencies, especially the authoritarian submission. Thus, the negative association between the drift rate and the likelihood of getting vaccinated was enhanced through authoritarian submission or obedience: information processing capacities were lower in individuals with higher authoritarian submission, who were more likely to delegate vaccination decision to CDC. Conversely, individuals with higher information processing capacity, especially those with lower obedience, were less likely to delegate decision-making and, possibly, were hesitant getting vaccinated. Importantly, the impacts of information processing capacities on vaccination decisions were independent from the impacts of trust in public institutions. Thus, preemptively building trust in public health institutions in individuals with higher information processing capacity, including those with lower tendencies toward obedience, could mitigate vaccine hesitancy.

Note that, the associations between authoritarian tendencies and perception of vaccine safety and vaccination decisions that we observe were predicted but should nevertheless be treated as preliminary, as we used only a subset of items from the F-scale, rather than the full validated scale. Future investigations should use the full F-scale and test these relationships more robustly.

We replicated prior findings^9–15,21,22,108^ that education, population density, and negative consequence of COVID positively associate with vaccine acceptance, while self-identified as Black was associated with vaccine hesitancy (significantly in the full sample). Some studies revealed important links between racial discrimination in the criminal justice system in vaccine hesitancy in Black individuals^109^, which could have reduced tendency to delegate decisions to government agencies. However, others suggested that access barriers played more important role^110^ and racial discrepancies in vaccination rates diminished over time^111^.

We additionally found important temporal dynamics. Perception of COVID vaccine safety improved over time. A positive perception of vaccine safety was increasingly associated over time with the decision to get vaccinated. Older age (a vaccination priority group) and being in later stage of pandemic (September–October 2021) robustly predicted higher vaccination likelihood. These associations were independent of prior vaccination status and are likely to reflect growing vaccine accessibility over time.

Given the complexity of modern medicine, with its ever-more opaque biomedical technologies, it is increasingly difficult to make informed medical decisions^112^. Thus, health decisions may become primarily a decision of *whom to trust*. We suggest that factors that influence such decisions should become a critical topic for future research on effectiveness of public health institutions. Rapidly developing medical innovations, such as mRNA vaccines, are likely to trigger strong skepticism in some individuals, including those with high authoritarian tendencies. The simple enforcement of mandatory vaccination by government institutions may facilitate compliance among these individuals, who exhibit high vaccination likelihood despite low perceived vaccine safety. Since the predisposition towards social conformity leads these individuals to identify with the existing social order^113^, establishing the CDC’s authority during times of stability may mitigate negative attitudes toward medical innovations during times of stress. Times of crisis tend to trigger elevated levels of paranoia^15^, which in turn corrode institutional trust (as our data illustrate). We suggest that public health institutions must accumulate enough trust during times of stability, so that people will follow their guidance during times of crisis.

Understanding vaccine hesitancy as a by-product of complex interactions between individual cognitive styles and how individuals relate to public health institutions, rather than viewing it as a “*new threat”*^114,115^ linked to particular socio-demographic groups^116^, can help to clarify mechanisms that underlie the phenomenon. We can adopt more effective policies by recognizing that trust, obedience to authority, and information processing capacity together contribute to vaccine hesitancy, as opposed to viewing the issue as predominantly political^60^. Future research may test whether the same key contributing factors impact the dynamics of public attitudes in other contexts, such as vaccine acceptance among parents of children with autism spectrum disorder^117^.

*Limitations* Our model did not fully address the role of mandates. We collected data via online self-reports; thus, verifying responses was beyond our control. Approximately 30% of the participants were recruited from Connecticut, and the rest from the Mechanical Turk (MTurk) subject pool, potentially limiting generalizability of our results to the U.S. population more broadly. While the Connecticut subsample was recruited to match the state population in age, gender, education, and income, it was not large enough to be representative of the state population. Combined with the MTurk convenience sample, it may not represent trends in the U.S. population more generally. Our sample primarily consists of participants located in high population density areas (Table 1), which is consistent with the population distribution in the U.S. but limits our ability to examine vaccine hesitancy in rural areas^25,26^.

Our longitudinal data included missing responses (Table S5). To preserve power, these responses were imputed. The PVDM task was completed by study participants in two contexts: 262 individuals completed the online task anonymously, while 56 completed the online task as a part of a larger study, during another phase of which they interacted with researchers in person. We did not see systematic differences in DDM parameters between these two groups (SI6, Figure S6, SI7, Table S7). Measures of paranoia and anxiety were only completed by 262 individuals from the “online only” sample this reduced the sample size for the respective analyses.

We treated the DDM parameters as temporally stable characteristics; while one-week test–retest reliability of these parameters was acceptable (SI8), longer term stability has not been established. To reduce the participants’ load, we only included 5 questions from the F-scale during the last wave of data collection*,* which significantly limits interpretability of our results. This approach of only using a subset of items has been successfully used before^76–78^. It resulted in a subscale of high reliability (Cronbach’s α = 0.82), and the authoritarian value orientation has been shown to be a temporally stable trait-level characteristic^118^. Despite being a key aspect of vaccine attitude, self-reported perceived safety does not capture every component of vaccine attitudes. We did not ask our participants about their political beliefs to avoid potential reporting biases. A variable state politics was based on the composition of the state legislature; this rough proxy is less sensitive than more granular measures of political ideology at the individual level.

Our SEM assumed that vaccine perceptions impact vaccine actions; however, the reverse is also possible—an instance of confirmation bias^119^.

## Methods

### Participants

All procedures were approved by Yale Institutional Review Board (IRB). Participants were recruited in 2019 for a study validating the PVDM task for online use (in preparation; SI6-SI8) and provided consent in accordance with IRB regulations and guidelines. Two samples were recruited:

1. A representative sample of the Connecticut population (N = 143) was recruited in person using flyers and online ads. This sample was age-matched to the state's age distribution, and within three age groups (18–35, 35–55, older than 50), participants were recruited to match race, ethnicity, education, and income distributions.
2. A sample (N = 531) recruited anonymously online through Mechanical Turk and local ads. Only participants whose responses to pre-screen questions (e.g., attention check questions, scores on the Lie scale greater than 7) passed rigorous quality control were enrolled.

All participants completed informed consent (online or in person) prior to data collection. All but 10 individuals consented to be re-contacted for future studies; in February 2021, these participants were invited to participate in a longitudinal follow up.

In 2021, 644 participants completed online surveys in three waves: February 20th to March 5th (N = 511), April 20th to May 5th (N = 475), and September 20th to August 15th (N = 502). 349 individuals completed all three waves. The final sample included 318 participants who reported vaccination status. In 2019, all 318 participants completed the PVDM task and a survey online; 262 participated anonymously (220 from Mechanical Turk, 42 from local ads) and 56 met researchers in person. For the purpose of the original study, initial assessments for the "online only" and "in person" groups used different instruments. Sample statistics are in Table 1. It should be noted that sampling partially from the local community and partially from the online convenience sample limits the data representativeness in the U.S.

### Measures

*Sociodemographic* measures (Table 1) include gender, race (Black/non-Black), ethnicity (Hispanic/non-Hispanic), age, income group, and level of education reported in 2019. We expected vaccine acceptance to positively associate with education and income, and negatively to being Black, Hispanic, and middle aged. We further expected male participants to be less likely to delegate the decision to CDC.

Participants reported the state of residence in 2021, which allowed us to derive two additional variables: state politics were determined based on 2021 data from the National Conference of State Legislature^104^; population density was based on 2021 data from U.S. Bureau of Labor Statistics^120^. Conservative political beliefs were expected to correlate with vaccine hesitancy. We intentionally did not include a question about our participants’ personal political beliefs, to avoid potentially being perceived as having a political agenda, which could have increased a reporting bias. Instead, we used the state politics as a very rough proxy. We expected population density to positively correlate with vaccine acceptance.

Negative COVID impact was assessed in each wave by two items: ‘Unemployed because of COVID? Yes/No’, ‘Have your family member(s) been infected with COVID? Yes/No/Not sure.’ It was expected to positively associate with a likelihood to get vaccinated^9,108^.

*Knowledge about COVID* was assessed in each wave by two questions from the CDC website ‘Frequently Asked Questions about COVID-19 Vaccines’ contemporary with each wave (e.g., “Can I get COVID from food? Yes/No”). Responses consistent with those from the CDC were coded as 1 and averaged across 2 questions. Knowledge about COVID could negatively associate with decision to delegate decision, but positively the level of trust in CDC and science, and a tendency to independently decide to get vaccinated.

In each wave of 2021, participants reported their perception of safety of COVID vaccines ("definitely no" = 1 to "definitely yes" = 7), how much science knows about COVID-19 vaccine (“nothing” = 1 to “a lot” = 7), trustworthiness of the CDC ("untrustworthy" = 1 to "trustworthy" = 5), and trustworthiness of social media, which was the average across TikTok, Twitter, YouTube, Instagram, and Facebook ("untrustworthy" = 1 to "trustworthy" = 7). Perception of vaccine safety was expected to positively associate with the likelihood of getting vaccinated; higher trust in the CDC and the science message were expected to positively correlate with the tendency to delegate the decision to the CDC; perceived trustworthiness of social media was expected to positively correlate with vaccine hesitancy^121^.

*Personality measures* were assessed in 2021, during wave 3. Internal (Cronbach’s α = 0.67) and external (Cronbach’s α = 0.81) locus of control (LOC) was measured with 8 items^77^ of the Rotter Locus of Control Scale^122^ ("strongly disagree" = 1 to "strongly agree" = 7). Internal locus of control was expected to negatively correlate with tendency to delegate the decision. The authoritarian orientation (Cronbach’s α = 0.81) was measured with 5 items from the F-scale^75^. It was expected to negatively correlate with trust in public health institutions, and positively with the tendency to comply with their recommendations. We have opted to use only a subset of questions from these two scales to reduce the subjects’ fatigue and to improve the completion rates; the selected items resulted in instruments with acceptable to good reliability. A similar approach was successfully used by prior studies that aimed to use these variables as covariates rather than to characterize nuances of the personality^33,76–78^. We acknowledge, that this limits interpretability of our results. Since the authoritarian orientation style is a multidimensional construct^25–31^, items were selected to represent different subscales relevant to the focus of the study: Authoritarian Submission (2 items) and Authoritarian Aggression (1 item); additionally, one Anti-interception item and one Superstition item were deemed to be highly relevant (see SI3). Selected items from both scales are reported in Tables S3-2 & S9.

The Brief Symptom Inventory (BSI**)** is a 53-item self-report instrument that assesses a broad range of psychiatric symptoms, including anxiety (Cronbach’s α = 0.95) and paranoid ideation (Cronbach’s α = 0.93). In 2019, participants from the ‘online only’ subsample rated how much they agree with each statement (e.g., “Feeling fearful”; “not at all” = 0 to “extremely” = 4). Scores higher than 1.7 for anxiety and 1.14 for paranoia are interpreted as clinically elevated^123^.

*Perceptual and value-based decision making (PVDM) task*^79^ On each trial, participants select one of two emotionally neutral grayscale images based on either (i) which image is darker (perceptual decision making, PDM), or (ii) which image they like better (value-based decision making, VDM). All trials were grouped in PDM and VDM blocks, 52 trials each. Correct choice in PDM is based on the objective darkness of each image. “Correct” choice in VDM is based on individual ‘liking’ ratings of each image, collected at the beginning and end of the first repetition of PDM and VDM trials. Following the second ‘liking’ ratings, PDM and VDM blocks were repeated (trials order randomized), but 25% of randomly selected trials followed with a short message on the screen “The choice is wrong” (PVD) or “The choice is inconsistent” (VDM). This manipulation was included to evaluate how negative affect^95–97^ impacts information processing style. Response time (RT) and accuracy are recorded on each of 208 trials to be analyzed using hierarchical Bayesian parameter estimation in the Drift Diffusion Model (HDDM)^124^. Trials with RT < 0.2 s and extreme outliers (RT < mean—3 SD or RT > mean + 3 SD) were discarded; the minimum number of remaining trials across participants was 180. HDDM allows simultaneously estimating the group level and the individual level parameters for each of the four conditions of the task. Our sample of 318 participants with at least 180 data points per participant was well powered for the planned analysis^125^. Decision boundary, drift rate, and non-response time are of interest to our study and used as predictors of perceived vaccine safety and vaccination action. Boundary decision measures response caution (i.e., the amount of evidence required to reach a decision); drift rate measures effectiveness of information processing; and non-response measures time spent encoding the stimulus and making the motor response. Measures of interest were DDM parameters in four task contexts.

### Missing data

In the full sample, measures of income, confidence in science competence, trusting CDC, knowledge level, and trusting social media contained less than 6% missing data; measures of authoritarian value orientation and locus of control contained less than 13% missing data. In the online sample, less than 7% of responses were missing (Table S5). Missing values were handled using a multiple imputation procedure with 5 imputations^126^. Because BSI was only administered to the ‘online only’ group, impacts of elevated anxiety and paranoia were only analyzed in the ‘online only’ subsample. This reduced the power of our analyses. Impacts of all other factors were first evaluated in the full sample and then confirmed in the ‘online only’ subsample.

### Data analysis

*Panel data analyses* were conducted, using NLogit 4.0^127^, to evaluate the impact of predictors of interest separately on perceptions of vaccine safety (linear model) and on the likelihood of getting vaccinated (logit model) during different stages of the vaccination program. Because vaccination decision is irreversible, vaccination status at time *t* depends on the vaccination status at time *t-1*; to account for that, the logistic regressions also included one period lagged vaccination status (lagged vaccination status was set to 0 in wave 1)^128,129^.

First, we fit a series of models to the full sample. The base model included predictors suggested by prior research (see Table 2):

Vaccination status/perceived safety ~ wave + gender + age group + race + ethnicity + education + income + income^2^ + COVID in family + unemployed because of COVID + state politics + population density + knowledge + science competence + trusting CDC + trusting social media + authoritarianism + internal LOC + external LOC

Model 1 also included DDM parameters: decision boundary, drift rate, and non-decision time, derived from VDM trials with random negative feedback. To evaluate specificity, follow up tests included these DDM parameters from the remaining three conditions, one at a time. Next, to evaluate whether impacts of some predictors variated across stages of the vaccination program, a predictor by waves (“ × *wave 1”* and “ × *wave 3”*) interactions were sequentially included in the model, one predictor at a time. Model 2 included all interaction terms that were significant at *p* ≤ 0.10 level, when entered individually. Finally, to preserve power, a short version of model 2 only included predictors from model 2 that were significant at *p* ≤ 0.10.

Next, we ran a series of models using the “online only” subsample. To evaluate any possible sampling biases, we first ran a short version of model 2. Next, model 3 also included elevated levels of anxiety and paranoia as binary predictors. Finally, to test whether cognitive distortions in anxiety and paranoia alter information processing, in model 4 we added interactions of remaining DDM parameters by elevated anxiety and elevated paranoia.

*Structural equation modeling (SEM)* using Amos 26, fit a model, depicted in Fig. 3, to data from wave 3 and the “online only” subsample. This model assumed that perception of vaccine safety influenced vaccination decisions, and that both depended on the level of trust in public health institutions and individual processing style. The model also assumed that individuals’ information processing styles (effectiveness of evidence accumulation quantified by the drift rate and cognitive distortions in elevated anxiety and paranoia) can influence individual attitude toward public institutions (trusting CDC and science more generally, and presence or lack of authoritarian tendencies) but were not influenced by them. Socio-demographic predictors from model 2 were included as control covariates. Additional post-hoc SEM was conducted with 5 individual items from F-scale instead of the average score to explore whether different dimensions of the authoritarian attributes relate differently to key variables of interest.

### Supplementary Information

Supplementary Information.

## Data Availability

The dataset analyzed during the current study is available at https://github.com/psychack/vaccinehesitancy.
