# Supplementary material for: Information processing style and institutional trust as factors of COVID vaccine hesitancy
Source: Sci Rep. 2024 May 6;14:10416. doi: 10.1038/s41598-024-60788-y (PMC11074285; doi:10.1038/s41598-024-60788-y)
Supplement: Supplementary file 1 — Supplementary Information. [file 41598_2024_60788_MOESM1_ESM.docx]

**Supplementary Materials**

**for**

**Information processing style and institutional trust as factors of COVID vaccine hesitancy**

**SI1. Descriptive statistics**

**Figure SI1.** Bivariate Pearson correlation among predictors.

**
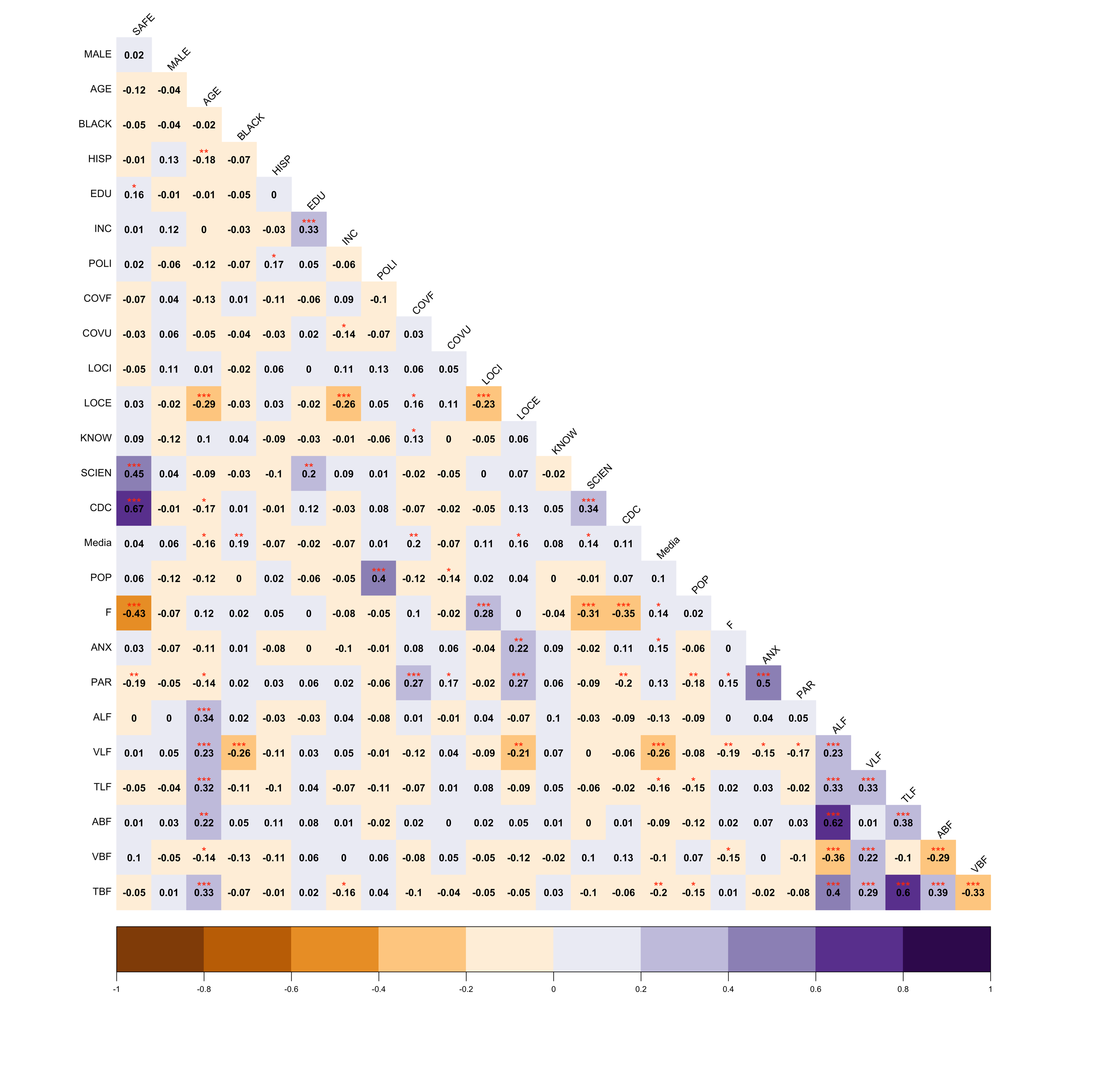
**

Correlation coefficients and significance levels between each variable, with non-responses dropped. N=213. * - p < 0.05, ** - p < 0.01, *** - p < 0.001. If measures were repeatedly evaluated across waves, we used their values at wave 3.

**Figure SI2.** Sample level changes over time**.**


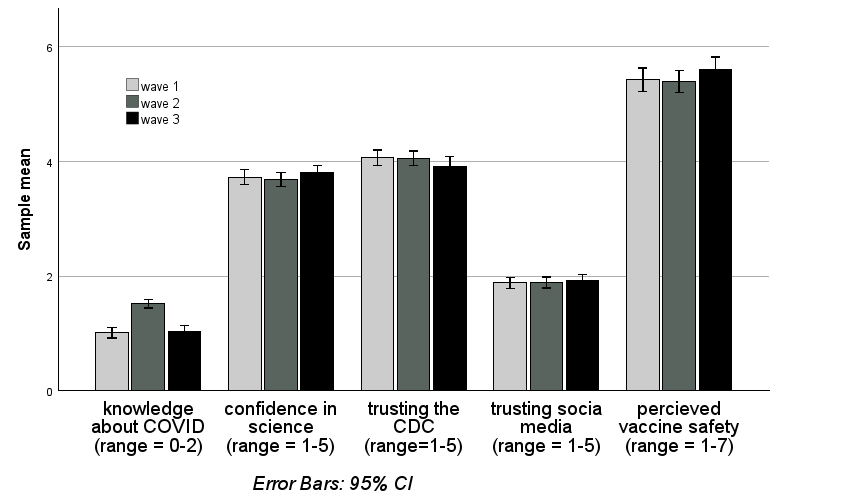


p < 0.05

p < 0.001

**Table S2-1.** Panel logistic regression model fit for DV=vaccinated

|  | Full sample (in-person & online) | | | | Online subsample | | |
| --- | --- | --- | --- | --- | --- | --- | --- |
|  | Base model | Model 1 | Model 2 | Model 2, short | Model 1 | Model 2 | Model 3 |
| Sensitivity | 0.80 | 0.80 | 0.81 | 0.80 | 0.79 | 0.79 | 0.80 |
| Specificity | 0.87 | 0.86 | 0.88 | 0.87 | 0.90 | 0.90 | 0.89 |
| Correct predictions | 0.83 | 0.83 | 0.85 | 0.84 | 0.84 | 0.85 | 0.84 |
| BIC | 0.91 | 0.92 | 0.97 | 0.88 | 0.88 | 0.88 | 0.89 |
| p-value | 0.36 | 0.22 | 0.28 | 6.00 | 4.89 | 0.78 | 0.94 |

Panel logistic regression model fit reflected by correctly predicted “vaccinated” (sensitivity), “not vaccinated” (specificity), overall correct predictions, Bayesian Information Criterion, and P value.

**Table S2-2.** Panel linear regression model fit for DV= Perceived Safety

|  | Full sample (in-person & online) | | | | Online subsample | | |
| --- | --- | --- | --- | --- | --- | --- | --- |
|  | Base model | Model 1 | Model 2 | Model 2, short | Model 1 | Model 2 | Model 3 |
| *X*^2^ | 457.71 | 466.13 | 475.57 | 464.38 | 373.41 | 345.35 | 351.85 |
| AIC | 0.99 | 1.00 | 1.00 | 0.97 | 1.04 | 0.99 | 0.98 |

Panel linear regression model fit reflected by Chi-square (*X^2^*) and Akaike Information Criterion. All p-values for *X*^2^ p< 0.01.

**SI3.** Exploratory Factor Analysis (EFA) with Principal Axis Factoring and Promax rotation with Kaiser Normalization was conducted to determine loadings of 5 selected items from F-scale. Principal Axis Factoring is more effective than Maximum Likelihood Factor in uncovering weaker factors^1^ and thus was preferable as a more conservative approach to detect multidimensionality. We selected Promax rotation because we allow the potential components to correlate.^2^ EFA revealed that all 5 items loaded onto one factor.

**Table S3-1.** Exploratory factor analysis on selected items from F-scale^3^: Eigenvalues and variance explained.

|  | | | | | | | |  |
| --- | --- | --- | --- | --- | --- | --- | --- | --- |
| Factor | Initial Eigenvalues | | | Extraction Sums of Squared Loadings | | | |  |
|  | Total | % of Variance | Cumulative % | Total | % of Variance | Cumulative % | |  |
| 1 | 2.897 | 57.936 | 57.936 | 2.380 | 47.607 | 47.607 | |  |
| 2 | .737 | 14.733 | 72.669 |  |  |  | |  |
| 3 | .543 | 10.856 | 83.525 |  |  |  | |  |
| 4 | .486 | 9.712 | 93.237 |  |  |  | |  |
| 5 | .338 | 6.763 | 100.000 |  |  |  | |  |
| **Table S3-2.** Exploratory factor analysis: Item loadings. | | | | | | | | |
| **Items** | | | | | | | **Factor 1** | |
| **F-scale q1 (*****Authoritarian submission*):** Obedience and respect are the most important virtues a child should learn | | | | | | | .75 | |
| **F-scale q2 (*Superstition*):** Science has its place, but there are many important things that can never be understood by the human mind | | | | | | | .74 | |
| **F-scale q4 (*Anti-intraception*):** Nowadays more and more people are prying into matters that should remain personal and private | | | | | | | .68 | |
| **F-scale q3 (*Authoritarian submission*):** Every person should have complete faith in some supernatural power whose decisions they obey without question | | | | | | | .67 | |
| **F-scale q5 (*****Authoritarian aggression*):** The true American way of life is disappearing so fast that force may be necessary to preserve it | | | | | | | .62 | |

**Note:** Selected items from F-scale^4^ were measured on from 1 (strongly agree) – 7 (strongly disagree).

**Table S4.** Structural equation modeling of standardized direct and indirect effects of variables of interest on decision to vaccinate by October 2021, with F-scale questions individually included as predictors.

|  | DV = Perception of vaccine safety | | | DV = Vaccinate | | | |  |
| --- | --- | --- | --- | --- | --- | --- | --- | --- |
|  | Direct | Indirect | Total | | Direct | Indirect | Total | |
| Perception of vaccine safety | - | - | - | | **0.56^****^ (0.08)** | - | **0.56^****^ (0.08)** | |
| *Measures of objective/observable factors* | | | | | | | | |
| Male | 0.06 (0.04) | - | 0.06 (0.04) | | -0.02 | 0.033 | 0.013 | |
| Age ≤ 35 years | -0.01 (0.05) | - | -0.01 (0.05) | | **0.12^**^ (0.05)** | -0.01 (0.03) | ***0.11^*^ (0.06)*** | |
| Age ≥ 60 years | 0.002 (0.04) | - | 0.002 (0.04) | | **0.12^****^ (0.04)** | 0.001 (0.02) | **0.13^***^ (0.05)** | |
| Black | -0.03 (0.05) | - | -0.03 (0.05) | | -0.07 (0.05) | -0.02 (0.03) | ***-0.09^*^ (0.05)*** | |
| Education level | **0.10^**^ (0.05)** | - | **0.10^**^ (0.05)** | | 0.06  (0.05) | **0.06^**^ (0.03)** | **0.12^**^ (0.05)** | |
| Income level | -0.23 (0.14) | - | -0.23 (0.14) | | -0.04 (0.13) | -0.13 (0.08) | -0.16 (0.16) | |
| Income level ^2^ | 0.18 (0.14) | - | 0.18 (0.14) | | 0.12  (0.13) | 0.10  (0.08) | 0.22  (0.16) | |
| State population density | 0.07 (0.05) | - | 0.07 (0.05) | | ***0.07^*^ (0.04)*** | 0.04 (0.03) | **0.11^**^ (0.05)** | |
| *Measures of subjective/unobservable factors* | | | | | | | | |
| Level of knowledge of science | **0.23^****^ (0.06)** | - | **0.23^****^ (0.06)** | | -0.03 (0.05) | 0.13 (0.04) | 0.09^*^ (0.06) | |
| Trust CDC | **0.42^****^ (0.06)** | - | **0.42^****^ (0.06)** | | **0.21^***^ (0.07)** | **0.24^****^ (0.05)** | **0.45^****^ (0.07)** | |
| **F-scale q1:** Obedience and respect are the most important virtues a child should learn | 0.002 (0.06) | - | 0.002 (0.06) | | -0.09^*^ (0.05) | 0.001 (0.03) | -0.09 (0.06) | |
| **F-scale q2:** Science has its place, but there are many important things that can never be understood by the human mind | **-0.18^***^ (0.06)** | - | **-0.18^***^ (0.06)** | | 0.02  (0.05) | **-0.10^***^ (0.04)** | -0.09 (0.06) | |
| **F-scale q3:** Every person should have complete faith in some supernatural power whose decisions they obey without question | 0.07 (0.07) | - | 0.07 (0.07) | | **0.25^****^ (0.05)** | 0.04  (0.04) | **0.29^****^**  **(0.07)** | |
| **F-scale q4:** Nowadays more and more people are prying into matters that should remain personal and private | -0.04 (0.07) | - | -0.04 (0.07) | | -0.05 (0.07) | -0.02 (0.04) | -0.08 (0.07) | |
| **F-scale q5:** The true American way of life is disappearing so fast that force may be necessary to preserve it | **-0.17^**^ (0.08)** | - | **-0.17^**^ (0.08)** | | 0.01  (0.07) | **-0.09^**^ (0.05)** | -0.08 (0.08) | |
| *Clinical measures* | | | | | | | | |
| Clinical level of anxiety | 0.03 (0.05) | 0.03 (0.05) | 0.06 (0.07) | | -0.03 (0.04) | 0.07  (0.05) | 0.04  (0.06) | |
| Clinical level of paranoia | -0.02 (0.06) | ***-0.09^*^ (0.05)*** | -0.11 (0.07) | | 0.01  (0.05) | -0.07 (0.05) | -0.06 (0.07) | |
| *Behavior-based measures of information processing* | | | | | | | | |
| Drift rate, value-based choices following emotional triggers | -0.01 (0.05) | -0.01 (0.04) | -0.02 (0.06) | | -0.06 (0.05) | -0.06 (0.05) | **-0.12^**^ (0.06)** | |

^a^ – p = 0.11, * - p < 0.1, ** - p < 0.05, *** - p < 0.01, **** - p<0.001; N = 272; **Model fit:** Ꭓ^2^(1)=0.32 p=0.57 CMIN/DF=0.32; Bootstrap K = 2000; RMSEA < 0.01 with PCLOSE = 0.68. **Indirect effect of paranoia on perception of vaccine safety** is through decreased trust in CDC message: Direct effect on trust in CDC β_std_ = -0.14 S.E = 0.07 p=0.05. **Indirect effect of paranoia on perception of vaccine safety** is through (i) decreased trust in CDC message (direct effect on trust in CDC β_std_ = -0.14 S.E = 0.07 p=0.05) and (ii) increased “conservatism” = higher score on F-scale q5 (direct effect on q5 β_std_ = 0.16 S.E = 0.06 p=0.02).

**Table S5.** Percentage of missing responses by variable, all were imputed.

|  | Full sample | online subsample |
| --- | --- | --- |
| Gender | 0.0% | 0.0% |
| Age | 0.9% | 0.8% |
| Race | 0.0% | 0.0% |
| Education | 0.0% | 0.0% |
| Income | 2.8% | 2.3% |
| Unemployed because of COVID | 3.8% | 4.2% |
| COVID in family | 4.7% | 5.0% |
| State of residence | 0.3% | 0.4% |
| Perceived knowledge of COVID (self) | 4.7% | 5.4% |
| Perceived knowledge of COVID (science) | 5.0% | 5.4% |
| Trust in CDC | 5.7% | 6.6% |
| Trust in social media | 8.2% | 6.9% |
| F-score | 12.9% | 6.6% |
| Internal locus of control | 12.9% | 6.6% |
| External locus of control | 12.9% | 6.6% |
| DDM measures | 0.3% | 0.4% |

**SI6.** Drift rate parameter is context dependent. It is affected by the type of the decision and by the negative emotions; but differently in different people.

**Figure SI6.** Drift rates in four experimental conditions of the PVDM task, by vaccination status in waves 1-2.


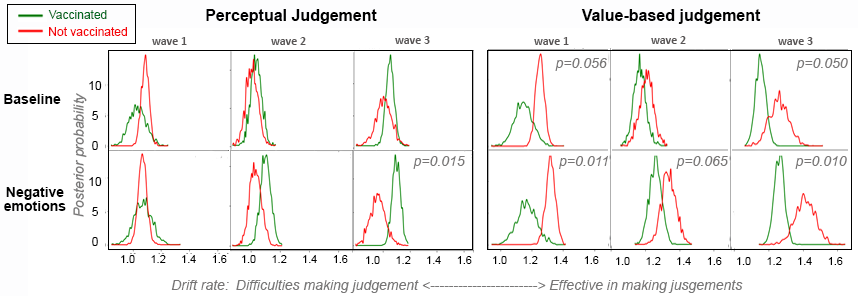


**SI7.** We used independent samples Mann-Whitney U test to compare distributions between two subsamples: collected in person and collected online across four experimental conditions. Since 12 tests were conducted, we set the significance level at p = 0.004 (equivalent to 0.05/12).

**Table S7.** Comparing distributions of DDM parameter between in-person and online subsample.

| **DDM parameters, by experimental conditions** | **Mann-Whitney U p-value** |
| --- | --- |
| Boundary (a), value-based choice, feedback | 0.20 |
| Drift rate (v), value-based choice, feedback | 0.29 |
| Non-decision time (t), value-based choice, feedback | 0.13 |
| Boundary (a), value-based choice, no feedback | 0.03 |
| Drift rate (v), value-based choice, no feedback | 0.07 |
| Non-decision time (t), value-based choice, no feedback | 0.29 |
| Boundary (a), perceptual choice, no feedback | 0.56 |
| Drift rate (v), perceptual choice, no feedback | 0.10 |
| Non-decision time (t), perceptual choice, no feedback | 0.95 |
| Boundary (a), perceptual choice, feedback | 0.22 |
| Drift rate (v), perceptual choice, feedback | 0.03 |
| Non-decision time (t), perceptual choice, feedback | 0.58 |

**SI8.** Test-retest reliability of DDM parameters from PVDM and validation for online administration.

As a part of another investigation (in preparation), we have collected both in-lab and on-line data in 148 individuals across a range of hardware configurations using both PVDM and RDM tasks. We used intraclass correlation reliability (ICC)^5^ coefficients to evaluate validity of DDM parameters recovered from the data collected online of the online. ICC greater than 0.5^6^ were deemed acceptable. In these analyses, we varied the number of trials included in the HDDM analyses to account for the possibility that participants become fatigued and are more likely to make careless choices by the end of each block. We find that most DDM parameters are recovered at a group level with acceptable ICCs (>0.54) using data from both tasks when the first 50 trials are included in the HDDM analyses. One exception was the boundary separation during perceptual easy choices (ICC=0.34); participants tended to rush during online sessions (reflected in significantly lower boundary separations). Thus, we used a version of PVDM that only included 50 trials in each condition for data collection.

**Table S9.** Selected items from Locus of Control^7^ scales

| **Locus of Control** |
| --- |
| Q1: How my life goes depends on me. |
| Q2: What a person achieves in life is above all a question of fate or luck. |
| Q3: I frequently have the experience that other people have a controlling influence over my life. |
| Q4: One has to work hard in order to succeed. |
| Q5: If I run up against difficulties in life, I often doubt my own abilities. |
| Q6: The opportunities that I have in life are determined by social conditions. |
| Q7: Inborn abilities are more important than any efforts one can make. |
| Q8: I have little control over the things that happen in my life. |

**Note:** Selected items from Locus of Control. F-scale were measured on from 1 (strongly agree) – 7 (strongly disagree).

**References:**

1 De Winter, J. C. & Dodou, D. Factor recovery by principal axis factoring and maximum likelihood factor analysis as a function of factor pattern and sample size. *Journal of applied statistics* **39**, 695-710 (2012).

2 Tabachnick, B. G., Fidell, L. S. & Ullman, J. B. *Using multivariate statistics*. Vol. 6 (pearson Boston, MA, 2013).

3 Adorno, T. *The authoritarian personality*. (Verso Books, 2019).

4 De Grazia, A. The Authoritarian Personality. By TW Adorno, Else Frenkel-Brunswik, Daniel J. Levinson, R. Nevitt Sanford.(New York: Harper & Brothers. 1950. Pp. xxxiii, 990. $7.50.). *American Political Science Review* **44**, 1005-1006 (1950).

5 Bliese, P. D. Group size, ICC values, and group-level correlations: A simulation. *Organizational research methods* **1**, 355-373 (1998).

6 Koo, T. K. & Li, M. Y. A guideline of selecting and reporting intraclass correlation coefficients for reliability research. *Journal of chiropractic medicine* **15**, 155-163 (2016).

7 Caliendo, M., Cobb-Clark, D. A. & Uhlendorff, A. Locus of control and job search strategies. *Review of Economics and Statistics* **97**, 88-103 (2015).
